# Effectiveness of a Tailored Fall-Prevention Program for Discharged Older Patients: A Multicenter, Preliminary, Randomized Controlled Trial

**DOI:** 10.3390/ijerph19031585

**Published:** 2022-01-30

**Authors:** Tetsuya Ueda, Yumi Higuchi, Gentoku Hattori, Hiromi Nomura, Gen Yamanaka, Akiko Hosaka, Mina Sakuma, Takato Fukuda, Takanori Fukumoto, Takashi Nemoto

**Affiliations:** 1 Graduate School of Comprehensive Rehabilitation, Osaka Prefecture University, Habikino City 583-8555, Osaka, Japan; yumi@rehab.osakafu-u.ac.jp; 2 Department of Rehabilitation, Yao Tokushukai General Hospital, Yao City 581-0011, Osaka, Japan; hatt.gentoku@gmail.com (G.H.); hiromi.nomura@tokushukai.jp (H.N.); 3 Department of Rehabilitation, Chiba-Nishi General Hospital, Matsudo City 270-2251, Chiba, Japan; reha@chibanishi-hp.or.jp; 4 Department of Rehabilitation, Hanyu General Hospital, Hanyu City 348-8505, Saitama, Japan; riha@fureaihosp.or.jp; 5 Department of Rehabilitation, Shonan Fujisawa Tokushukai Hospital, Fujisawa City 251-0041, Kanagawa, Japan; iam.mina37@gmail.com; 6 Department of Rehabilitation, Uji Tokushukai Medical Center, Uji City 611-0041, Kyoto, Japan; uji-pt@ujitoku.or.jp; 7 Department of Rehabilitation, Nozaki Tokushukai Hospital, Daito City 574-0074, Osaka, Japan; nozareha@tokushukai.jp; 8 Department of Rehabilitation, Shonan Kamakura General Hospital, Kamakura City 247-8533, Kanagawa, Japan; takashi.nemoto@tokushukai.jp

**Keywords:** fall prevention, discharged patients, home floor plans, multicenter, acute-care hospital, intervention study

## Abstract

This multicenter, preliminary, randomized controlled trial investigated the effect of a tailored fall-prevention program using home floor plans for discharged orthopedic patients aged ≥65 years who experienced ≥1 fall(s) in the past year (*n* = 72) at five acute-care hospitals. The control group received standard care (exercise to prevent recurrent falls), whereas the intervention group received a tailored fall-prevention program in addition to usual care. A physical therapist conducted the tailored education program using each patient’s home floor plans before discharge. A follow-up survey of falls and near-falls at home was performed using a monthly fall calendar for the 1-month period after discharge. Data on 81.5% of participants remained for the final analyses. No falls occurred in the intervention group; however, 4.3% of those in the control group experienced a fall. Near-falls were reported by 3.7% and 26.9% of the participants in the intervention and control groups, respectively. The proportion of participants who did not experience near-falls in the 1st month after discharge was lower in the intervention than in the control group (*p* = 0.018). In conclusion, the tailored fall-prevention program using home floor plans in multiple acute-care hospitals was effective in reducing falls and near-falls in discharged orthopedic patients.

## 1. Introduction

Older patients often fall at their homes after being discharged from hospitals [1,2,3,4,5]; thus, it may take longer for them to recover functions, including physical mobility and activities of daily living. Several strategies have been suggested to prevent falls in older people [6,7,8,9,10,11,12,13,14,15,16,17,18,19]. Over recent years, there have been increasing reports on fall-prevention measures for discharged patients [1,3]. However, no effects were observed after using a fall-prevention multimedia education package [1], as well as a physical training tailored program including follow-up on vision, polypharmacy, and environmental hazards [3]. Therefore, although reports on fall-prevention measures are increasing, no effects have been observed in discharged patients.

Similar to prior studies, we conducted a fall-prevention program using home floor plans for discharged older patients [20]. Our program was proven to be effective in preventing falls during the 1-month follow-up period. Recently, the effects of fall-prevention interventions based on external factors, such as environmental hazards, have attracted attention [6,15,16,21]. Additionally, our study reported favorable results regarding a fall-prevention intervention for discharged patients. However, this result could not be generalized, as the patients were from a single facility. Therefore, we needed to determine the effect of the fall-prevention program using home floor plans across multiple facilities.

We conducted this study to investigate the effect of a tailored fall-prevention program using home floor plans for discharged older patients in multiple acute-care hospitals.

## 2. Materials and Methods

### 2.1. Trial Design

A multicenter, parallel, preliminary, randomized controlled trial with equal allocation to the intervention and control groups was conducted among individuals aged ≥65 years. This study was conducted at five acute-care hospitals throughout Japan. The five hospitals were Chiba-Nishi General Hospital in Chiba, Hanyu General Hospital in Saitama, Shonan Fujisawa Tokushukai Hospital in Kanagawa, Uji Tokushukai Medical Center in Kyoto, and Nozaki Tokushukai Hospital in Osaka. The supporting Consolidated Standards of Reporting Trials checklist is available as supporting information. The staff members who conducted interviews, performed assessments, and received calendars from participants were unaware of the group allocation.

### 2.2. Participants

**Figure 1:**
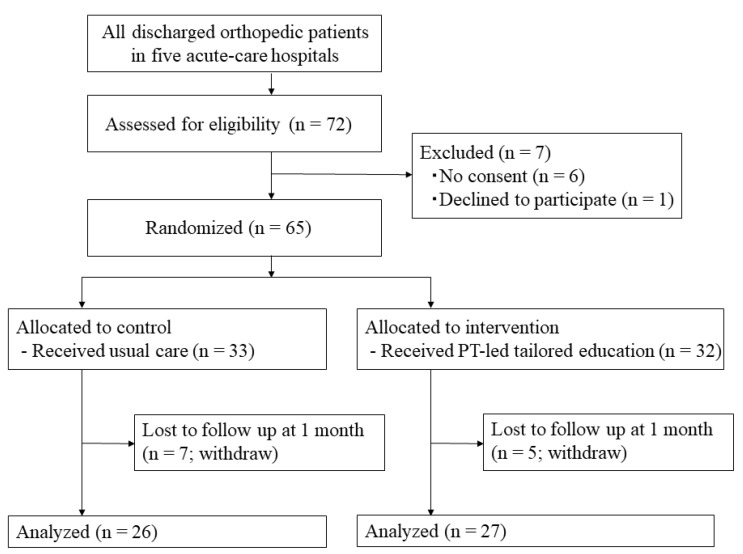
Flow chart of participants.

The participants were adults aged ≥65 years who were admitted to the orthopedic ward of five acute-care hospitals, had a history of falls in the past year, and had been discharged with the ability to walk indoors. The participants were recruited between November 2017 and January 2019; 72 patients participated during the study period. We chose this sample size for this study because similar sample sizes have been used in previous preliminary studies [12,20]. We excluded patients with cognitive impairment, defined as a Mini-Mental State Examination score of <18 points (*n* = 0); patients who spoke little Japanese or could not speak the Japanese language (*n* = 0); patients with severe neurological and/or visual disorders (*n* = 0); patients planning to move within the next month (*n* = 0); patients who could not get consent (*n* = 6), and patients who declined to participate (*n* = 1) (Figure 1). The purpose of this study was explained to all patients before obtaining written consent. This study was conducted in accordance with the principles embodied in the 1975 Declaration of Helsinki (as revised in 2013) and was conducted with approval from the Research Ethics Committee of the Mirai Iryo Research Center (approval number: TGE00912-004; date of approval: 4 October 2017). Informed consent was obtained from all individuals involved in the study; written informed consent was obtained from the patients to publish this paper. The study was registered at the University Hospital Medical Information Network Clinical Trials Registry (UMIN-CTR; UMIN000029798).

### 2.3. Randomization

Stratified randomization was performed using a computer-generated random number schedule. To ensure blinded randomization, the randomization schedule was generated in advance and was only accessible to a blinded member of staff not involved in participant recruitment, interviews, or assessments. Following baseline assessment and after obtaining informed consent and approval for trial participation from the individuals, the research staff contacted the blinded member of the staff verbally to allocate individual participants to their respective groups.

### 2.4. Interventions

The tailored education program to prevent falls described in this study is similar to our previously reported program [20]. Participants were randomized into one of the following groups: the intervention group, which received a tailored education program using home floor plans, and the control group, which received usual care (exercise to prevent recurrent falls). A physical therapist conducted the program using home floor plans drawn by patients prior to hospital discharge. A staff training program was developed for a few skilled physical therapists. It consisted of a lecture by senior staff regarding home fall hazards based on the 2011 American Geriatrics Society and British Geriatrics Society guidelines [15]. The staff training program included instructions on educating patients to prevent falls.

**Figure 2:**
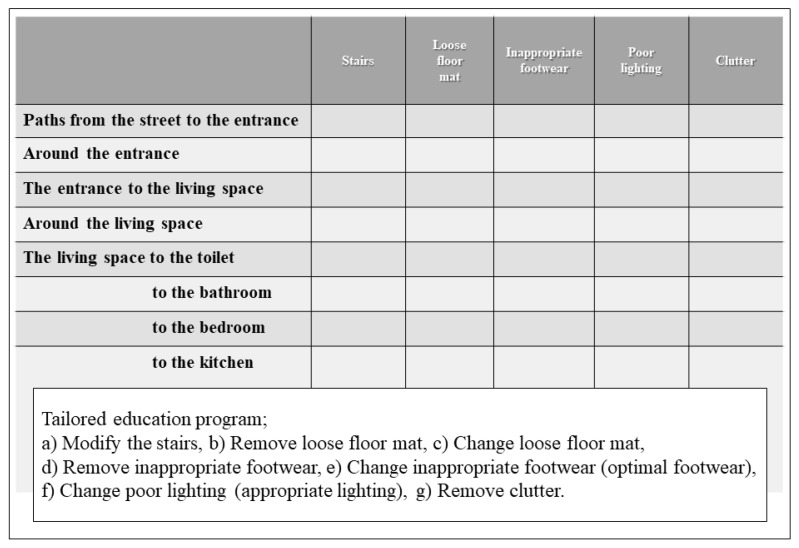
Checklist for avoiding home fall hazards based on the home floor plans.

First, we checked the paths used during daily living at the homes of individuals based on their home floor plans, such as the paths from the street to the entrance; the entrance to the living space, and the living space to the toilet, bathroom, bedroom, and kitchen. Second, using the home floor plans, we confirmed home fall hazards during individual face-to-face interviews. We asked them about the following to clarify whether there were home fall hazards in the paths that the participants used frequently during daily life: whether there were any stairs, whether the floors in the living room and bedroom were clear, whether floor mats were held in place (so they would not slide), whether the participants wore footwear that fit poorly or had high heels, and whether there was poor lighting placement [6,15,16]. Third, we conducted a tailored education program regarding modifying the stairs, clearing away clutter, removing or changing loose floor mats, wearing optimal footwear, and ensuring appropriate lighting. We created a checklist to ensure that the interventions could be performed at all relevant areas in the patient’s home (Figure 2). We administered usual care by providing exercise to prevent recurrent falls in the intervention and control groups.

### 2.5. Baseline Assessment

A baseline evaluation was performed 3 days before discharge from the hospital; cognitive function, activities of daily living, physical function, and mental and psychological function were evaluated. Evaluations were performed using the Barthel Index for activities of daily living, the Timed Up and Go test for physical function, the Geriatric Depression Scale 5, and the Modified Fall Efficacy Scale for mental and psychological function.

Additionally, data regarding the participants’ characteristics (i.e., age, sex, body mass index, primary diseases, comorbidities, and medication status), fall injury causing hospitalization, number of falls in the past year, living environment, house environment, certification for long-term care before admission, sedentary time before hospitalization, walking ability before and after hospitalization, and length of hospital stay were collected from their medical records.

### 2.6. Follow-Up

A follow-up survey of falls and near-falls at home was performed at 1 month after discharge from the hospital. Near-falls were defined as a slip, trip, or loss of balance (i.e., the individual starts to fall, but can stop or prevent the fall to the ground or a lower surface) [22]. Information regarding falls and near-falls was collected using a monthly fall calendar, which was sent to the subjects through mail. An interview via phone was conducted for those who could not return the calendar.

### 2.7. Statistical Analysis

All analyses were conducted using an intention-to-treat principle. Two participants (one each in the intervention and control groups) who did not provide any follow-up data were treated as missing. To compare the baseline characteristics between the intervention and control groups, unpaired t-tests and chi-square tests were used for continuous and categorical variables, respectively. The time elapsed between hospital discharge and the first near-fall event was analyzed using Kaplan–Meier curves and compared using the log-rank test. All statistical analyses were conducted using SPSS Statistics version 25 (IBM Corporation, Armonk, NY, USA). Statistical significance was set at *p* < 0.05.

## 3. Results

### 3.1. Participants’ Characteristics

A total of 65 patients were enrolled in this study (Figure 1). Seven and five participants in the control and intervention groups, respectively, withdrew from the study. During the 1-month follow-up period, 12 participants were lost to follow-up and were withdrawn from the study; therefore, 53/65 (81.5%) participants remained for the final analyses.

The demographic characteristics of the participants are presented in Table 1. There were no significant differences in the baseline characteristics between the intervention and control groups. Overall, there were more lower limb diseases than upper limb diseases. The walking ability was lower at discharge than before hospitalization; however, the level of independence was high as a result of the Barthel Index score. Both the Barthel Index score and participant age were more favorable in the intervention than in the control group; however, there were no significant differences in each group. All participants had undergone rehabilitation, and when they were discharged from the hospital, they were able to walk independently using at least walking aids. At discharge, 33% and 43.8% of the participants in the control and intervention groups, respectively, could walk without walking aids.

### 3.2. Falls (Primary Outcome)

No falls occurred in the intervention group (*n* = 27) during the follow-up period (Table 2). However, one participant (4.3%) in the control group (*n* = 26) experienced a fall during the 1-month follow-up period. In the participant who fell at home, there was no injury due to the fall.

### 3.3. Near-Falls (Secondary Outcome)

**Figure 3:**
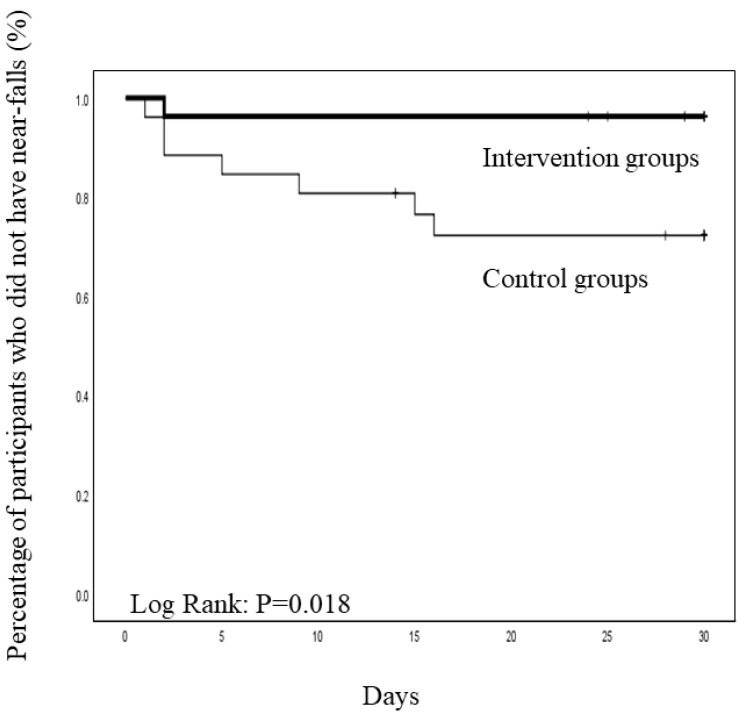
Kaplan–Meier analysis of the proportion of patients who did not experience near-falls in the first month after discharge. Seven patients in the control group and one patient in the intervention group recorded near-falls.

Near-falls were reported by one participant (3.7%) in the intervention group and seven participants (26.9%) in the control group during the study period (Table 2). Between-group comparisons of the incidence of near-falls within the 1-month period are presented in Figure 3. Kaplan–Meier analysis revealed that the proportion of patients who did not experience near-falls in the 1st month after discharge was lower in the intervention than in the control group (*p* = 0.018).

## 4. Discussion

In this study, the effect of a tailored fall-prevention program using home floor plans was investigated in discharged older patients aged ≥65 years across multiple acute-care hospitals. The major finding was that the tailored fall-prevention program in multiple facilities was effective against falls and near-falls during the 1-month follow-up period after discharge.

Our program to prevent falls has been previously reported to be effective in a single hospital [20]. In this follow-up study, we assessed the fall-prevention effects of the program in five hospitals. To the best of our knowledge, there are no reports of a fall-prevention effect achieved by fall-prevention programs including only external factors, such as environmental hazards, among discharged older patients aged ≥65 years. Although several studies have been conducted in this field [1,3], no fall-prevention effect has been observed. In contrast, our program was effective in preventing falls, which we believe is because we checked the paths taken during daily living at the individuals’ homes based on the home floor plans provided by them.

No falls occurred in the intervention group during the follow-up period. The same results were obtained for both the effect in a single facility and the effect in multiple facilities. We presume that the participants of this study had yet to sufficiently recover their physical functions at 1 month after discharge. We consider that an educational fall-prevention intervention program focused on the environmental factors that contribute to falls was effective in complementing the physical function of the participants during the early stages of post-discharge recovery. Therefore, we believe that our study has sufficient clinical significance.

Near-falls were significantly prevented in our tailored fall-prevention program using home floor plans during the 1-month follow-up period. We hypothesize that this method of approaching external factors was useful because it was performed at a time when the physical function had not improved dramatically for the participants in this study. The recognition of near-falls is critical for preventing falls, as near-falls may lead to falls [23,24,25]. As more falls have been reported during the 1st month after discharge [2,5], it was very significant that we could prevent near-falls during this follow-up period. Further research regarding near-falls is necessary to prevent falls in older people after discharge.

This study had some limitations. First, although this study was conducted across multiple acute-care hospitals, the number of participants and the number of falls were both small. We believe that a larger-scale, randomized controlled trial is needed in the future. Second, we were unable to clarify whether the falls in this study were caused by environmental factors. However, as the participants in this study had yet to sufficiently recover their physical functions after discharge, we believe that internal factors also had a considerable impact on the occurrence of falls. Third, the design of this trial was single-blinded, which may have introduced bias; the intervention group might have been more likely to under-report falls and near-falls. Fourth, this study did not provide information on all diseases commonly found in older patients, such as orthostatic hypotension, arrythmias, syncope, Parkinson’s disease, and alcoholism. However, it included diseases, such as hypertension, diabetes mellitus, chronic obstructive pulmonary disease, heart disease, stroke, and musculoskeletal disease similar to those in previous studies.

## 5. Conclusions

The tailored fall-prevention program using home floor plans in multiple acute-care hospitals was effective in reducing falls and near-falls in discharged orthopedic patients. In future studies, we believe that this program should be considered to prevent falls in older patients after discharge.

## Figures and Tables

**Table 1:** Baseline characteristics and assessment items of study participants according to the control group and the intervention group.

	Control Group (n = 33) Mean ± SD	Intervention Group (n = 32) Mean ± SD	*p*-Value
**Age (years)**	77.9 ± 6.6	75.1 ± 6.8	0.095 ^†^
**Sex (female), *n* (%)**	25 (75.8)	22 (68.8)	0.528 *
**Body mass index (kg/m^2^)**	21.8 ± 3.5	22.6 ± 3.6	0.385 ^†^
**Primary diseases, *n* (%)**			0.783 *
**Upper limb disease**	2 (6.1)	2 (6.3)	
**Lower limb disease**	23 (69.7)	22 (68.8)	
**Trunk disease**	7 (21.2)	8 (25.0)	
**Upper and lower limb disease**	1 (3.0)	0 (0)	
**Comorbidities, *n* (%)**			
**Hypertension**	15 (45.5)	17 (53.1)	0.536 *
**Diabetes mellitus**	8 (24.2)	10 (31.3)	0.528 *
**Chronic obstructive pulmonary disease**	0 (0)	0 (0)	1.000 *
**Heart disease**	12 (36.4)	7 (21.9)	0.199 *
**Stroke**	2 (6.1)	2 (6.3)	0.682 *
**Musculoskeletal disease (aside from primary disease)**	5 (15.2)	7 (21.9)	0.485 *
**Medication status, *n* (%)**			
**Psychotropic drug**	0 (0)	0 (0)	1.000 *
**Benzodiazepine**	1 (3.0)	0 (0)	0.508 *
**Antidepressant**	1 (3.0)	0 (0)	0.508 *
**Anticonvulsant**	0 (0)	0 (0)	1.000 *
**Fall injury causing hospitalization, *n* (%)**	30 (90.9)	29 (90.6)	0.649 *
**Number of falls in the past year (*n*)**	1.5 ± 1.8	1.8 ± 2.2	0.514 ^†^
**Living environment: alone, together, alone daytime (*n*)**	10/19/4	6/20/6	0.494 *
**House environment: detached house (*n*)**	25 (75.8)	27 (84.4)	0.385 *
**Certification for long-term care before admission, *n* (%)**	6 (18.2)	3 (9.4)	0.253 *
**Sedentary time before hospitalization (hours/day)**	8.2 ± 5.5	6.4 ± 4.1	0.154 ^†^
**Walking ability prior to admission: walking alone, *n* (%)**	28 (84.4)	29 (90.6)	0.372 *
**Length of hospital stay (days)**	29.2 ± 15.0	26.3 ± 13	0.397 ^†^
**Functional evaluation at discharge**			
**Barthel Index (score)**	89.2 ± 11.3	93.4 ± 8.4	0.095 ^†^
**Timed Up and Go test** **(secs)**	17 ± 8.6	15.9 ± 13.3	0.708 ^†^
**Geriatric Depression Scale 5 (score)**	1.1 ± 1.3	0.8 ± 0.8	0.408 ^†^
**Modified Fall Efficacy Scale** **(scores)**	90.3 ± 36.7	95 ± 34.7	0.599 ^†^
**Walking ability at discharge: walking along, *n* (%)**	11 (33)	14 (43.8)	0.388 *

Abbreviation: SD, standard deviation. For the Barthel Index, the score range is 0–100; for Geriatric Depression Scale 5, the score range is 0–5); for the Modified Fall Efficacy Scale, the score range is 0–140. * Chi-square test. ^†^ Unpaired *t*-test.

**Table 2:** Falls and near-fall circumstances of study participants according to each group.

	Control Group (*n* = 26)	Intervention Group (*n* = 27)	*p*-Value
**Participants who fell, *n* (%)**	1 (4.3)	0 (0)	
**Total falls, *n***	1	0	
**Number of injuries due to falls, *n***	0	0	
**Participants who nearly fell, *n* (%)**	7 (26.9)	1 (3.7)	0.022 *
**Total near-falls, *n***	42	1	0.175 ^†^

* Chi-square test. ^†^ Unpaired *t*-test.

## Data Availability

The data presented in this study are available on request from the corresponding author. The data are not publicly available due to restrictions regarding privacy or ethics.
